# Detection and molecular characterisation of a diagnosis escape variant associated with occult hepatitis B virus in Brazil

**DOI:** 10.1590/0074-02760160477

**Published:** 2017-07

**Authors:** Ricardo Wagner de Almeida, Francisco Campello do Amaral Mello, Isabelle Vasconcelos Menegoy, Márcia Paschoal do Espírito Santo, Cléber Ferreira Ginuíno, Paulo Sérgio Fonseca de Sousa, Livia Melo Villar, Elisabeth Lampe, Lia Laura Lewis-Ximenez

**Affiliations:** Fundação Oswaldo Cruz-Fiocruz, Instituto Oswaldo Cruz, Laboratório de Hepatites Virais, Rio de Janeiro, RJ, Brasil

**Keywords:** occult hepatitis B infection, HbsAg, genotype, mutation

## Abstract

**BACKGROUND:**

Many studies have identified mutations in the hepatitis B surface antigen (HBsAg) as important factors limiting the ability of commercial serological assays to detect this viral antigen. However, an association between mutations in the HBsAg gene and the occurrence of occult HBV infection (OBI) in patients has not been established.

**OBJECTIVES:**

To detect hepatitis B virus (HBV) DNA in patients with anti-HBc as a unique serological marker, a previously published, cost-effective TaqMan-based real-time polymerase chain reaction (PCR) test with minor groove binding probes was adapted for use in this study. The current study also aimed to investigate HBsAg mutations and genotypes of HBV in OBI at the Viral Hepatitis Ambulatory Clinic in Rio de Janeiro to determine any possible association.

**METHODS:**

Intra-assay and inter-assay reproducibility were determined, and the mean coefficient of variation values obtained were 2.07 and 3.5, respectively. Probit analysis indicated that the 95% detection level was 25 IU/mL. The prevalence of OBI was investigated in 35 serum samples with an ‘anti-HBc alone’ profile from individuals who attended our clinic between 2011 and 2013.

**FINDINGS:**

HBV DNA was detected in only one sample, resulting in an OBI rate of 2.9%. Nucleotide sequencing of the pre-S/S region was performed to genotype and analyse mutations within the HBsAg gene of this HBV DNA. The HBV in the OBI case was classified as sub-genotype A1, and a sequence analysis of the small S gene revealed 12 mutations in the major hydrophilic region compared to the consensus A1 sequence. Most of these mutations occurred in amino acid residues that have been reported as clinically relevant because they have been implicated in vaccine escape and/or inability to detect HBsAg by commercial serological assays.

**MAIN CONCLUSIONS:**

Our study suggests the importance of specific HBsAg mutations, different from those in D, B, and C genotypes, in sub-genotype A1 HBV associated with OBI.

Hepatitis B virus (HBV) is a major cause of acute and chronic hepatitis, which represents a serious threat to public health worldwide. According to the World Health Organization (WHO), chronic infection caused by HBV affects approximately 350 million people worldwide. Viral hepatitis B and C are the leading causes of cirrhosis and hepatocellular carcinoma (HCC), despite the availability of a safe and effective hepatitis B virus vaccine (Liaw & Chu 2009).

Occult HBV infection (OBI) is often defined as the presence of HBV DNA in serum/plasma without hepatitis B surface antigen (HbsAg) that is detectable in clinical practice (Hollinger & Sood 2010, Cheng et al. 2014). OBI can be divided into two categories, based on hepatitis B markers: a group that is negative for all hepatitis B serological markers and a group that expresses antibodies against HBV core antigen (anti-HBc) as the only detectable serological marker. This ‘anti-HBc alone’ profile is apparent after the disappearance of HBsAg and before the appearance of anti-HBs antibodies. This anti-HBs window usually lasts a few weeks. ‘Anti-HBc alone’ can also represent a false positive result for anti-HBc. However, most people who test positive for ‘anti-HBc alone’ will continue to test positive for ‘anti-HBc alone’ for years or even decades (Zeinab 2011), suggesting that this serological profile is not limited to a specific window period (the recovery phase of an HBV infection) or indicative of delayed immunity. Such cases are often interpreted as a ‘low dose’ HBV infection or an atypical infection with a mutant HBV that is poorly defined (Grob et al. 2000). As a result, the finding of ‘anti-HBc alone’ cannot be clearly interpreted to determine prognosis or infectivity. The question regarding the treatment of cases with ‘anti-HBc alone’ is controversial, especially for those individuals who also test positive for circulating HBV DNA (Knöll et al. 2006). Most experts do not recommend any treatment, because these cases usually have normal liver enzyme levels. However, individuals with ‘anti-HBc alone’ might be epidemiologically important as infectious HBV carriers, and specific precautions might be necessary to prevent viral dissemination (Lledó et al. 2011).

Although HBV DNA detection in serum by quantitative real-time polymerase chain reaction (qRT-PCR) assay is the gold standard for identification of HBV infection, HBsAg is considered the main serological marker for diagnosis by routine laboratory tests (Reddy et al. 2011). The presence of mutations, including deletions, substitutions, or residue insertions, in the primary sequence of the surface (S) protein can affect its antigenicity, hindering or preventing HBsAg detection by anti-HBs antibodies and leading to false negative results in commercial assays. Amino acid substitutions from glycine to arginine at residue 145 (G145R) (Lacombe et al. 2013) or lysine to glutamic acid at residue 141 (L141E) (Karthiguesu et al. 1994) and in other ‘a’ determinant positions (Ma & Wang 2012) have this effect.

In this study, we aimed to identify OBI in ‘anti-HBc alone’ individuals, adapting a previously described real-time PCR assay for HBV DNA quantification (Weinberger et al. 2000). We also analysed mutations in the HBsAg and basal core promoter (BCP)/pre-core region that might contribute to the occult character of HBV detected in the present study.

## MATERIALS AND METHODS

*Ethics approval* - The study was conducted in accordance with the Declaration of Helsinki, and the protocol was approved by the Fiocruz Ethics Committee under protocol number 575/10.

*Study population* - We attempted to detect HBV DNA in 35 plasma samples from individuals that were previously characterised as having anti-HBc as the only serological marker (‘anti-HBc alone’) of HBV infection. Sera from all ‘anti-HBc alone’ cases were also tested for other serological markers of HBV (HBsAg and anti-HBs) and HCV (anti-HCV) infection. All serological markers were examined using the ARCHITECT system (Abbott Diagnostics, Delkenheim, Germany). In addition, 10 plasma samples from healthy individuals (those seronegative for all hepatitis serological markers) were tested as negative controls to confirm the specificity of the qRT-PCR assay.

Serum and plasma samples were collected at the Viral Hepatitis Ambulatory Clinic in Rio de Janeiro, Brazil, from 2011 to 2013 and were stored at -70ºC. This clinic serves as the National Reference Centre for Hepatitis Viruses in Brazil and receives persons suspected of having viral hepatitis, including acute and chronic cases and their contacts.

*DNA extraction* - To isolate HBV DNA from plasma, a High Pure Viral Nucleic Acid Kit (Roche Diagnostics, Mannheim, Germany) was used, per manufacturer’s instructions. DNA was extracted from 200 μL of plasma, with the addition of 1 μL of internal control (1× IPC DNA; Applied Biosystems, Foster City, CA, USA), and eluted in a final volume of 50 μL.

*Primers and probe modification for the qRT-PCR assay* - Primers HBV-Taq 1 and HBV-Taq 2 and probe BS-1 were designed from conserved regions of the HBV surface gene by using Primer Express software supplied by the manufacturer of the TaqMan Sequence Detection System (Perkin-Elmer-Applied Biosystems, Foster City, CA) as previously described (Weinberger et al. 2000). These primers sequences were identical to sequences in 95% of all published HBV genomes (as contained in GenBank release 110.0, 12/98). In contrast with the original fluorescent probe that was 5′-labelled with FAM and 3′-labelled with TAMRA, we modified the chemistry of the probe to 5′ NED and 3′ MGB-NFQ (primer and probe sequences are provided in Table I). To ensure the reliability of the assay with this probe modification, parameters of the qRT-PCR assay were evaluated and an international HBV panel was used for validation.

**TABLE I:** Oligonucleotides sequences used as primers and probe (10) in quantitative real-time polymerase chain reaction (qRT-PCR) assay

Oligo	Function	Sequence (5’-3’)	Position^*a*^
HBV-Taq 1	TaqMan PCR forward primer	CAA CCT CCA ATC ACT CAC CAA C	321-342
HBV-Taq 2	TaqMan PCR reverse primer	ATA TGA TAA AAC GCC GCA GAC AC	401-379
BS-1	TaqMan PCR probe	NED-TCC TCC AAT TTG TCC TGG TTA TCG CT-**MGB NFQ** ^*^	349-374

a: nucleotide position according to Genbank entry X51970 (20); *: modified probe.

*qRT-PCR* - A PCR and fluorogenic probe hybridisation master mix of reagents was prepared, containing 1 µL of SuperScript III RT/Platinum Taq Mix, 25 µL of 2× Reaction Mix (a buffer containing 0.4 mM each dNTP and 6 mM MgSO_4_), 1 µL of ROX Reference Dye, 300 nM oligonucleotide primers HBV-Taq1 and HBV-Taq2, 200 nM TaqMan probe BS-1, and nuclease free water to a final volume of 38 µL. A TaqMan 7300 system was used to perform the assay as described by the manufacturer. As an internal control, we used TaqMan Exogenous Internal Positive Control Reagents with 1× IPC Mix (primers and TaqMan probe labelled with VIC) and 1× IPC DNA. Amplification was performed in a 50-µL reaction mixture containing 10 µL of template DNA or nuclease-free water (control). We used SuperScript III RT/Platinum with the intent to later include primers and probes for HCV in a multiplex assay. All samples were assayed in duplicate. Amplification reactions began with an incubation step at 95ºC for 10 min and a PCR cycling program consisting of 50 two-step cycles of 30 s at 95ºC and 60 s at 60ºC, for a total run of about 2 h and 10 min.

*Preparation of the standard and calculation of the limit of detection (LOD)* - To generate a standard curve, serial dilutions of plasma with an HBV DNA load of 2.5-10^6^ IU/mL that were previously tested with Cobas AmpliPrep/Cobas TaqMan HBV v2.0 were used. For each run, triplicate samples of the dilutions were analysed. A linear regression of mean threshold cycles (Ct) values as a function of the decadic logarithm of the number of template molecules per reaction was performed. The values reported for the unknown samples were relative to the standard used.

HBV DNA values were calibrated using an international HBV reference panel developed by BBI Diagnostics (West Bridgewater, MA, USA). The HBV DNA linearity panel PHD801 consists of one negative and nine positive samples at concentrations of 2.4 × 10^6^, 6.9 × 10^5^, 1.2 × 10^5^, 5.2 × 10^4^, 2.0 × 10^4^, 1.7 × 10^3^, 1.4 × 10^2^, and 1.4 × 10^2^ IU/mL, and two HBV genotype A samples at less than 100 IU/mL.

*Statistical analysis* - For the reproducibility analysis, viral loads were summarised as standard deviations (SD) and coefficients of variation (CV). All analyses were performed in Excel. Probit analysis was performed to determine the LOD of the TaqMan PCR assay.

*Nucleotide sequencing* - The sample found to be positive for HBV DNA by qPCR assay was submitted to nucleotide sequencing of the pre-S/S and pre-core/core regions. The pre-S/S region was amplified by semi-nested PCR using primers and conditions described by Mello et al. (2012). The pre-core/core region was also amplified by semi-nested PCR using primer pairs X4 (5′-AAGGTCTTACATAAGAGGAC-3′, nt positions 1644-1663) and C2 (5′-CTAACATTGAGATTCCCGAGATTGAGA-3′, 2432-2458) in the first round and X5 (5′-ACTCTTGGACTCBCAGCAATG-3′, 1662-1682) and C2 in the second round. In both rounds, DNA amplification was performed with 5 U/µL Taq DNA polymerase (Invitrogen, San Diego, CA, USA), 10 mM dNTPs, 10× PCR buffer, 50 mM MgCl_2_, and 10 µM each primer pair in a final volume of 50 µL, under the following conditions: one cycle at 94ºC for 3 min, 30 cycles at 94ºC for 30 s, 55ºC for 30 s, and 72ºC for 1 min 30 s, and a final elongation step (7 min at 72ºC). PCR products were purified using a Wizard SV Gel and PCR Clean-Up System (Promega, Madison, WI, USA) and prepared for sequencing using a BigDye Terminator 3.1 Cycle Sequencing Kit (Applied Biosystems). The following primers were used: internal sense primers PS4 (5′-ACACTCATCCTCAGGCCATGCAGTG-3′, 3194-3218) and S4 (5′-TGCTGCTATGCCTCATCTTCT-3′, 416-436) and antisense primers PS8 (5′- TTCCTGAACTGGAGCCACCA-3′, 63-82) and SR (5′- CGAACCACTGAACAAATGGC-3′, 685-704) for the pre-S/S region; and internal sense primers C1 (5′-CTGTGGAGTTACTCTCGTTTTTGC-3′, 1935-1958) and C5 (5′- AGACCACCAAATGCCCCTATC-3′, 2299-2319) and antisense primers C3 (5′- TTGCCTGAGTGCAGTATGGT-3′, 2056-2075) and C4 (5′- CTTTATACGGGTCAATGTCCA-3′, 1902-1922) for the pre-core/core. Sequencing reactions were analysed on an ABI 3730 automated sequencer (Applied Biosystems).

*Phylogenetic and mutational analysis* - HBV genotyping was performed by phylogenetic analyses of the pre-S/S and pre-core/core genes of the virus identified in this study and HBV sequences representing all genotypes available in GenBank. Phylogenetic analyses were performed using the maximum likelihood method (bootstrap resampling with 1,000 replicates) in MEGA version 6.0 under the GTR+G+I nucleotide substitution model that was selected as the best-fit model by jModelTest (Posada 2008).

To identify amino acid substitutions in the small S gene and pre-core and BCP regions that could contribute to the occult character of HBV infection, 50 small S and 31 pre-core/core sequences from GenBank were compared to that of our HBV DNA.

## RESULTS

*qRT-PCR assay parameters* - *Reproducibility* - Inter-assay variability was determined by performing the assay on three consecutive days, and intra-assay variability was evaluated by testing three replicates for each sample. Mean viral loads, SD and CV are shown in Table II.

**TABLE II:** Reproducibility of quantitative real-time polymerase chain reaction (qRT-PCR) assay for hepatitis B virus (HBV) detection

	n times	Ct value observed Mean (SD)	CV (%)
Intra-assay			
6 log_10_	3	24.92 (0.45)	1.83
5 log_10_	3	26.94 (0.46)	1.71
4 log_10_	3	30.12(1.32)	4.39
3 log_10_	3	25.2 (0.37)	1.46
2 log_10_	3	44.45 (1.03)	2.32
1 log_10_	3	47.07 (0.62)	1.31
0 log_10_	3	47.185 (0.71)	1.51
Inter-assay			
6 log_10_	3	25.1 (0.55)	2.18
5 log_10_	3	28.41 (0.95)	3.33
4 log_10_	3	32.68 (2.10)	6.43
3 log_10_	3	39.61 (1.19)	3.02
2 log_10_	3	43.72 (1.27)	2.91
1 log_10_	3	46.62 (1.50)	3.23
0 log_10_	3	47.87 (1.60)	3.34

Ct: threshold cycles; CV: coefficients of variation; SD: standard deviation.

**Fig. 1:**
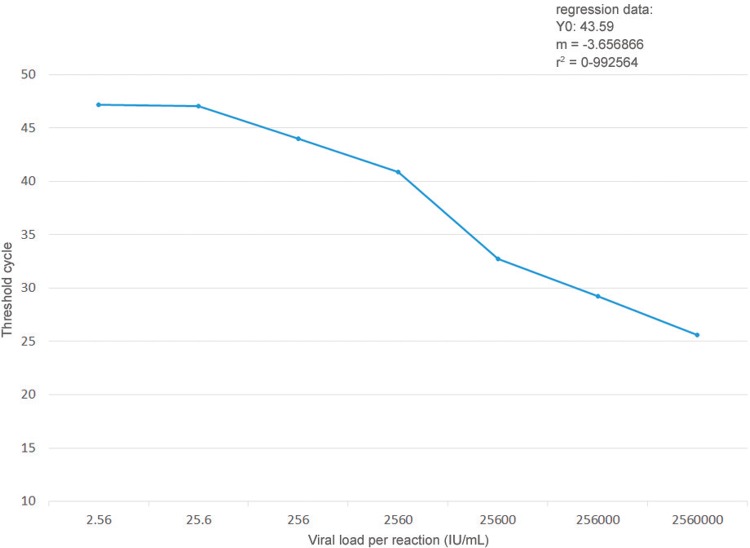
: mathematical ﬁtting of serial dilutions of a plasma sample positive for hepatitis B virus (HBV) DNA with a viral load of 6.40 log10 IU/mL. The threshold cycle (average from triplicate samples ± standard deviation) is plotted against the decadic logarithm of the viral load in each reaction. Linear regression produced a theoretical ordinate value for one template molecule (y0), the gradient in each cycle per order of magnitude (m), and a coefﬁcient of reliability (r2) close to the optimum of 1.

*Sensitivity and specificity* - Serial dilutions of an HBV DNA sample (2.56-10^6^ ‘UI’/mL) were amplified three times by qRT-PCR according to the above protocol. The Ct values were plotted against the dilutions to generate a standard curve. These values were proportional to the logarithm of the input copy number over six orders of magnitude (R^2^ = 0.99). The calculated PCR efficiency for this assay, based on a slope of -3.65, was 0.90 (Fig. 1).

We tested, in duplicate, 10 samples from healthy individuals, and none had detectable HBV DNA. Negative controls (NC), one for every 20 samples tested, were included in every reaction, and they were consistently found negative.

*Clinical sensitivity* - The clinical sensitivity of the qRT-PCR assay was determined using a low-titre dilution series of an HBV genotype A specimen. HBV DNA was diluted to concentrations of 2500, 250, 25, and 2.5 IU/mL. Seven independent assays were performed, with each dilution tested in triplicate, yielding 21 results for each DNA concentration. The 95% LOD of the assay, calculated by Probit analysis, was 25 IU (Table III).

**TABLE III:** Probit analysis used to determine the 95% detection limit of the hepatitis B virus (HBV) real time polymerase chain reaction (PCR) assay using a genotype A specimen

Measured HBV DNA concentration IU/mL	N° (%) of positive wells
2500	21/21
250	21/21
**25**	**20/21**
2.5	17/21

The assay clinical sensitivity was calculated using 15 replicate measurements at each dilution obtained from five independent experiments. The data used for the analysis are shown in regular typeface, and the calculated 95% detection limit of 25 IU/mL is shown in boldface.

*Validation with an international panel* - The HBV DNA PHD801 linearity panel was used, with one negative and nine positive samples assessed. Each panel sample was tested following the same procedure used for unknown samples. The results showed variation of less than 0.5 log_10_ IU/mL when comparing the observed and expected values. Variation of less than 0.5 log_10_ RNA/DNA copies number/mL 0.5 log_10_ RNA/DNA IU/mL is generally considered acceptable in molecular assays (Baleriola et al. 2011).

*Determination of OBI in ‘anti-HBc alone’ samples by qRT-PCR* - Of 35 individuals with an ‘anti-HBc alone’ profile, nine (25.7%) were female and 26 (74.3%) were male. Their ages ranged from 16 to 76, with a mean age of 49.85. HBV DNA was detected in one of 35 ‘anti-HBc alone’ individuals, indicating an OBI rate of 2.9% in our ambulatory care clinic between 2011 and 2013.

**Fig. 2:**
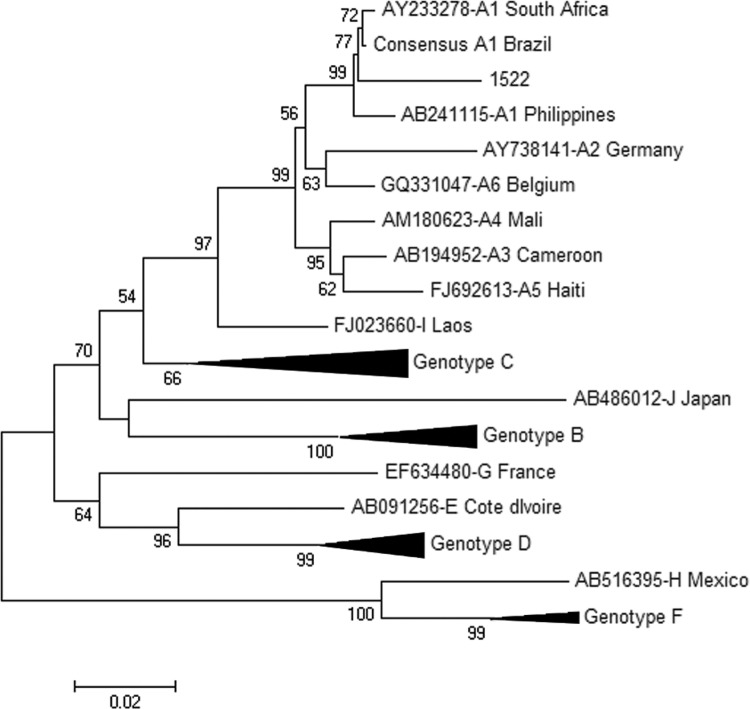
: maximum likelihood phylogenetic tree illustrating the relationship between pre-S/S region sequences of various hepatitis B virus (HBV) genotypes and the occult HBV infection (OBI) sequence obtained in this study (sample 1522; indicated with a dot). The classification of the study sample (genotype A, sub-genotype A1) was confirmed by phylogenetic analysis of the pre-core/core region (tree not shown). Bootstrap values are shown at tree nodes; non-A sub-genotypes were compressed to condense tree information.

*Genotyping and sequence analysis of the OBI sample* - Two genomic regions, pre-S/S and pre-core/core, of the qRT-PCR-positive sample (sample id: 1522) were PCR amplified and nucleotide sequenced to confirm the presence of HBV DNA and, thus, the occult character of this infection. To determine the genotype of HBV in the sample, a phylogenetic analysis of the pre-S/S region was performed that included representative HBV sequences from all known HBV sub-genotypes. The HBV from this OBI case was classified as genotype A, sub-genotype A1 (Fig. 2).

A calculation of genetic distances between sequences of the pre-S/S region in sub-genotype A1 (d = 0.03) and between sequences of this region in the A1 group and OBI sample (d = 0.041) revealed that, although being classified as sub-genotype A1, the OBI sample had many more nucleotide substitutions than those in other A1 sequences. Most of these appeared in the major hydrophilic region (MHR) of HBsAg and have been found contribute to non-detection of this antigen by commercial serological assays.

**Fig. 3:**
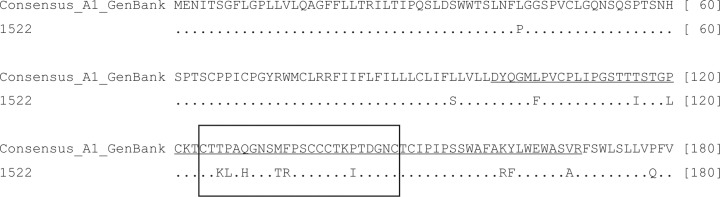
: amino acid sequence alignment of hepatitis B virus (HBV) partial small S protein comparing the occult HBV infection (OBI) isolate from this study (1522) with a consensus sequence of 50 HBV sub-genotype A1 sequences retrieved from GenBank. Amino acid positions are shown in brackets at the end of each line, and the MHR, amino acids 99-169, is underlined in the consensus sequence. The ‘a’ epitope is shown in a dashed box.

An amino acid sequence analysis of the small S gene of sample 1522 revealed 15 substitutions relative to the A1 consensus sequence, most (12/15) of which were located in the MHR of HBsAg, which spans residues 99-169 (Fig. 3). Within this region, the following substitutions were observed: L104F, T116I, P120L, T126K, P127L, Q129H, M133T, F134R, P142I, K160R, Y161F, and V168A. The other three substitutions, found outside MHR, were L42P, L94S, and P178Q.

**Fig. 4:**
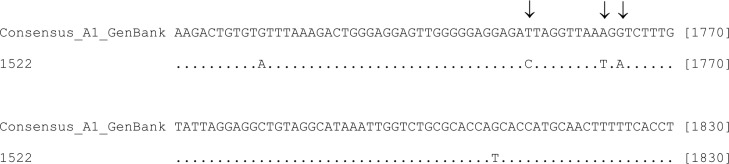
: nucleotide sequence alignment comprising the partial hepatitis B virus (HBV) basal core promoter and pre-core region. Comparison of the occult HBV infection (OBI) sequence obtained in the current study (1522) with a consensus sequence of 39 HBV sequences retrieved from GenBank. Nucleotide positions are shown in brackets at the end of each line, and substitutions A1762T, G1764A, and T1753C are indicated by arrows.

In the pre-core/core region, sample 1522 had a double substitution (A1762T/G1764A) in the BCP and substitution T1753C that has been shown to alter HBV replication *in vitro* (Fig. 4).

## DISCUSSION

Based on the reported high sensitivity (LOD of ~ 50 HBV genome equivalents), ability to detect all eight HBV genotypes by targeting the conserved regions of the HBV S gene, and widespread use internationally, we elected to introduce the qRT-PCR assay published by Weinberger et al. (2000) into our diagnostic laboratory, with some modifications. Initially, we decided to modify the probe at its 3′ end, replacing the TAMRA quencher with MGB-NFQ. This was done because TAMRA, in contrast with MGB-NFQ, is not a dark quencher, and its native fluorescence can interfere with FAM fluorescence, thus increasing the background fluorescence and limiting the sensitivity of the probe. The evaluation of parameters in our qRT-PCR assay indicated that this modification of the probe did not alter the quality or reliability of the test.

To create a standard curve, we used a plasma sample with an HBV DNA load of 6.40 log_10_, previously quantified on a commercial automated platform (Cobas AmpliPrep/Cobas TaqMan HBV Test, v2.0). We also included IPC DNA in these samples, and its presence did not interfere with construction of the standard curve.

To validate the assay, we used the HBV DNA PHD801 linearity panel, consisting of a negative sample and nine positive samples. Samples in this panel were tested as unknowns, and the results obtained were compared with the expected results. No sample showed variation greater than 0.5 log_10_, which is in the acceptable range for results of molecular tests (Baleriola et al. 2011). Our assay showed a sensitivity of at least 25 IU/mL, which is appropriate for investigating cases of OBI.

The use of nucleic acid tests (NATs) has decreased the risk of HBV transmission through blood transfusions. Where HBV DNA testing is not available, such as in developing countries, anti-HBc can be used as a less than ideal surrogate marker of possible seropositive OBI individuals, when blood, tissue, or organs are donated or when immunosuppressive therapy must be administered. However, the cost effectiveness and availability of NATs should be considered before clinical application (Kwak & Kim 2014). Our participation in external quality assessment and proficiency testing programs remains necessary to assess, validate, and improve the quality of our in-house technology.

This is the first time our clinic has investigated cases of individuals with an ‘anti-HBc alone’ profile. We aimed to establish a cost-effective qRT-PCR test to detect HBV DNA in our ambulatory patients and identify genomic mutations in OBI cases that could contribute to the hidden character of these infections. Commercial tests commonly use a sample volume of 1,000 µL, whereas our in-house qRT-PCR assay only used 200 µL. This can minimise the risks associated with blood sample collection and reduce the number of samples rejected because of insufficient volumes. Although the benefits of automated extraction are considerable, there are potential drawbacks. It is most economical when instruments are fully loaded to justify the capital investment that is required.

Previously, Barros Jr et al. (2008) assessed the occurrence of OBI in 51 patients with an anti-HBc-positive and HBsAg-negative profile in the Brazilian Amazon Region. Patients were tested by PCR, and 17% were found to exhibit this profile. Alencar et al. (2008) evaluated the presence of OBI in patients with HCV-related liver cirrhosis, with or without HCC, in São Paulo, and only one case with HCC had HBV DNA-positive serum.

De Matos et al. (2013) investigated the prevalence of OBI in 149 HBsAg-negative injecting drug users in the Central-West region of Brazil. Of these individuals, 19 were found positive for HBV DNA, resulting in an OBI prevalence of 12.7%. Six of these 19 individuals had anti-HBs and/or anti-HBc antibodies. These findings suggest a high prevalence of OBI in the Central-West region of Brazil. Franz et al. (2013) also investigated the prevalence of OBI, testing 207 HBsAg-negative kidney transplant recipients. HBV DNA, indicating OBI, was detected in two patients (1%).

Moresco et al. (2014) investigated the prevalence of OBI in the state of Amazonas, Brazil. Among 3,600 blood donations, 799 (22.2%) were anti-HBc reactive. The results showed that a small percentage of anti-HBc-reactive donors carried HBV DNA (2.7% vs. 2.9% reported here), and some of these specimens had anti-HBs titres above 100 mIU/mL, indicating the concomitant presence of the virus and high antibody titres, suggesting the possibility of HBV transmission from asymptomatic donors, especially in areas of high endemicity.

Our ambulatory care clinic reported 83 HbsAg-positive results in 2012. Individuals with an ‘anti-HBc alone’ profile were not reported to the municipal health department. Our findings suggest that all ‘anti-HBc alone’ cases should be investigated using sensitive molecular techniques, and if HBV DNA is detected, these cases should be immediately reported.

Our ultimate goal was to study OBI-associated HBV mutations, especially in the MHR, which comprises amino acids 99-169 and is involved in binding of antibodies to HBsAg. The ‘a’ determinant is within the MHR, comprising amino acids 124-147. The antibodies found in vaccinated individuals and antibodies used in immunoassays for HBsAg are directed against this ‘a’ determinant. Mutations in the ‘a’ determinant may affect the antigenicity of HBsAg and cause false negative results in some commercial tests for HBsAg (Lazarevic 2014).

Svicher et al. (2012) conducted a study to determine the molecular basis of occult HBV infection and found 20 mutations in the major hydrophilic region of HBV genotype D strains that strongly correlated with OBI. Our study of HBV genotype A1 DNA showed 12 mutations in the MHR. T126K, P127L, Q129H, M133T, F134R, and P142I occurred in the ‘a’ determinant, in the same position as in genotype D with other amino acids. The remaining eight mutations surrounding the ‘a’ determinant (L104F, T116I, P120L, K160R, Y161F, and V168A) were completely different from the mutations reported to be associated with OBI in HBV genotypes B, C, and D (Yuan et al. 2010), suggesting that some OBI-associated mutations are unique to a HBV genotype.

The results of a study by Carman et al. (1990) suggest that the loss of proline at position 120 significantly reduces the binding of antibodies to that region. Baclig et al. (2014) reported that a methionine-to-leucine substitution at position 133 (M133L) has no effect on protein structure. However, if a mutation to threonine occurs in the same position (M133T), it has a significant impact on HBsAg, compromising its detection. Both of these mutations were found in the genotype A1 HBV in our study.

Other substitutions in the ‘a’ determinant are considered vaccine escape mutations. The G145R mutation, which is frequently detected worldwide, was absent in our OBI-associated genotype A1 HBV. However, other substitutions in the ‘a’ determinant that have been associated with escape from vaccine-induced immunity were found in the genotype A1 sample obtained in this study at positions 116, 120, 126, 129, 133, and 142 (Chong-Jin et al. 1999, Lee et al. 2001).

Our study of an HBV genotype A1 strain showed T1762, A1764, and T1753 mutations in the BCP region. In vivo and in vitro studies have shown that mutations occurring in the BCP region modulate HBeAg expression at the transcriptional level. The typical double mutation in the BCP, A1762T/G1764A, is responsible for decreased pre-core mRNA synthesis (Xie et al. 2015). Experiments with site-directed mutants revealed that a 1753/1762/1764 triple mutant mildly reduced HBeAg expression, similar to the 1762/1764 mutant. Thus, core promoter mutations other than those at positions 1762 and 1764 can have major impacts on viral DNA replication and HBeAg expression (Parekh et al. 2003).

In conclusion, our study suggests the association between HBsAg mutations in sub-genotype A1 HBV, which are different from those described in genotypes D, B, and C, and OBI that test negative by currently available tests. HBsAg is a sentinel marker used in blood bank donor screening to prevent transmission of HBV to patients receiving transfusions. An understanding of reactivity of immunoassays with HBsAg mutants is important for establishing an appropriate testing algorithm for HBV detection programs. Specific sequence characteristics may be associated with high hepatitis B e-antigen (HBeAg) negativity and low HBV DNA levels in carriers of this HBV sub-genotype.

## References

[B1] Alencar RSM, Gomes MMS, Sitnik R, Pinho JRR, Malta FM, Mello IMVGC (2008). Low occurrence of occult hepatitis B virus infection and high frequency of hepatitis C virus genotype 3 in hepatocellular carcinoma in Brazil. Braz J Med Biol Res.

[B2] Baclig M, Alvarez M, Gopez-Cervantes J, Natividad F (2014). Unique surface gene variants of hepatitis B virus isolated from patients in the Philippines. J Med Virol.

[B3] Baleriola C, Johal H, Jacka B, Chaverot S, Bowden S, Lacey S (2011). Stability of hepatitis C virus, HIV, and hepatitis B virus nucleic acids in plasma samples after long-term storage at -20 degrees C and -70 degrees C. J Clin Microbiol.

[B4] Barros GM, Braga WSM, de Oliveira CMC, Castilho MC, Araújo JR (2008). Occult hepatitis B: prevalence and clinical characteristics in a population with high endemicity of hepatitis B infection in the western Brazilian Amazon Region. Rev Soc Bras Med Trop.

[B5] Carman WF, Zanetti AR, Karayiannis P, Waters J, Manzillop G, Tanzi E (1990). Vaccine-induced escape mutant of hepatitis B virus. Lancet.

[B6] Cheng HR, Kao JH, Wu HL, Chen TC, Tseng TC, Liu CH (2014). Clinical and virological features of occult hepatitis B in patients with HBsAg seroclearance post-treatment or spontaneously. Liver Int.

[B7] Chong-Jin O, Ning CW, Shiuan K, Keow LG (1999). Identification of hepatitis B surface antigen variants with alterations outside the “a” determinant in immunized Singapore infants. J Infect Dis.

[B8] de Matos MAD, Ferreira RC, Rodrigues FP, Marinho TA, Lopes CLR, Novais ACM (2013). Occult hepatitis B virus infection among injecting drug users in the Central-West Region of Brazil. Mem Inst Oswaldo Cruz.

[B9] Franz C, Perez RM, Zalis MG, Zalona ACJ, Rocha PTMCA, Gonçalves RT (2013). Prevalence of occult hepatitis B virus infection in kidney transplant recipients. Mem Inst Oswaldo Cruz.

[B10] Grob P, Jilg W, Bornhak H, Gerken G, Gerlich W, Günther S (2000). Serological pattern “anti-HBc alone”: report on a workshop. J Med Virol.

[B11] Hollinger FB, Sood G (2010). Occult hepatitis B virus infection: a covert operation. J Viral Hepat.

[B12] Karthiguesu V, Allison L, Fortuin M, Mendy M, Whittle HC, Howard CR (1994). A novel hepatitis B virus in the sera of immunized children. J Gen Virol.

[B13] Knöll A, Hartmann A, Hamoshi H, Weislmaier K, Jilg W (2006). Serological pattern “anti-HBc alone”: characterization of 552 individuals and clinical significance. World J Gastroenterol.

[B14] Kwak MS, Kim YJ (2014). Occult hepatitis B virus infection. World J Hepatol.

[B15] Lacombe K, Boyd A, Lavocat F, Pichoud C, Gozlan J, Miail-hes P (2013). High incidence of treatment-induced and vaccine-escape hepatitis B virus mutants among human immunodeficiency virus/hepatitis B-infected patients. Hepatology.

[B16] Lazarevic I (2014). Clinical implications of hepatites B virus mutations: recent advances. World J Gastroenterol.

[B17] Lee KM, Kim YS, Ko YY, Yoo BM, Lee KJ, Kim JH (2001). Emergence of vaccine-induced escape mutant of hepatitis B virus with multiple surface gene mutations in a Korean child. J Korean Med Sci.

[B18] Liaw YF, Chu CM (2009). Hepatitis B virus infection. Lancet.

[B19] Lledó JL, Fernández C, Gutiérrez ML, Ocaña S (2011). Management of occult hepatites B virus infection: an update for the clinician. World J Gastroenterol.

[B20] Ma Q, Wang Y (2012). Comprehensive analysis of the prevalence of hepatitis B virus escape mutations in the major hydrophilic region of surface antigen. J Med Virol.

[B21] Mello FCA, Lago BV, Lewis-Ximenez LL, Fernandes CA, Gomes SA (2012). Detection of mixed populations of wild-type and YMDD hepatitis B variants by pyrosequencing in acutely and chronically infected patients. BMC Microbiology.

[B22] Moresco MNS, Virgolino HA, Morais MPE, Motta-Passos I, Gomes-Gouvea MS, Assis LMS (2014). Occult hepatitis B virus infection among blood donors from the Brazilian Amazon: implications for transfusion policy. Vox Sanguinis.

[B23] Parekh S, Zoulim F, Ahn SH, Tsai A, Li J, Kawai S (2003). Genome replication, virion secretion, and e antigen expression of naturally occurring hepatitis B virus core promoter mutants. J Virol.

[B24] Posada D (2008). jModelTest: phylogenetic model averaging. Mol Biol Evol.

[B25] Reddy PB, Mukherjee RM, Aparna J, Mitnala S, Rao PN, Gupta R (2011). Detection of diagnosis escape variants of Hepatitis B virus by in house polymerase chain reaction assay. J Gen Mol Virol.

[B26] Svicher V, Cento V, Bernassola M, Neumann-Fraune M, Hemert FV, Chen M (2012). Novel HBsAg markers tightly correlate with occult HBV infection and strongly affect HBsAg detection. Antiviral Res.

[B27] Weinberger KM, Wiedenmann E, Bohm S, Jilg W (2000). Sensitive and accurate quantitation of hepatitis B virus DNA using kinetic fluorescence. J Virol Methods.

[B28] Xie Y, Liu S, Zhao Y, Zhang L, Zhao Y, Liu B (2015). Precore/core region mutations in hepatitis B virus DNA predict postoperative survival in hepatocellular carcinoma. PLoS ONE.

[B29] Yuan Q, Ou SH, Chen CR, Ge SX, Pei B, Chen QR (2010). Molecular characteristics of occult hepatitis B virus from blood donors in southeast China. J Clin Microbiol.

[B30] Zeinab NAS (2011). An overview of occult hepatitis B virus infection. World J Gastroenterol.

